# Ultrasound in Adhesive Capsulitis: A Narrative Exploration from Static Imaging to Contrast-Enhanced, Dynamic and Sonoelastographic Insights

**DOI:** 10.3390/diagnostics15151924

**Published:** 2025-07-31

**Authors:** Wei-Ting Wu, Ke-Vin Chang, Kamal Mezian, Vincenzo Ricci, Consuelo B. Gonzalez-Suarez, Levent Özçakar

**Affiliations:** 1 Department of Physical Medicine and Rehabilitation, National Taiwan University Hospital, Bei-Hu Branch, Taipei 10845, Taiwan; wwtaustin@yahoo.com.tw; 2 Department of Physical Medicine and Rehabilitation, National Taiwan University College of Medicine, Taipei 100233, Taiwan; 3 Center for Regional Anesthesia and Pain Medicine, Wang-Fang Hospital, Taipei Medical University, Taipei 110301, Taiwan; 4 Department of Rehabilitation Medicine, First Faculty of Medicine and General University Hospital in Prague, Charles University, 12800 Prague, Czech Republic; kamal.mezian@gmail.com; 5 Physical and Rehabilitation Medicine Unit, Luigi Sacco University Hospital, ASST Fatebenefratelli-Sacco, 20133 Milano, Italy; vincenzo.ricci58@gmail.com; 6 Research Center for Health Science, University of Santo Tomas, Manila 1008, Philippines; bebetsuarez61@gmail.com; 7 Department of Physical Medicine and Rehabilitation, Our Lady of Lourdes Hospital, Manila 1016, Philippines; 8 Department of Physical and Rehabilitation Medicine, Hacettepe University Medical School, Ankara 06100, Turkey; lozcakar@yahoo.com

**Keywords:** frozen shoulder, sonography, contrast-enhanced ultrasonography, dynamic examination, sonoelastography

## Abstract

Adhesive capsulitis is a painful and progressive condition marked by significant limitations in shoulder mobility, particularly affecting external rotation. Although magnetic resonance imaging is regarded as the reference standard for assessing intra-articular structures, its high cost and limited availability present challenges in routine clinical use. In contrast, musculoskeletal ultrasound has emerged as an accessible, real-time, and cost-effective imaging modality for both the diagnosis and treatment guidance of adhesive capsulitis. This narrative review compiles and illustrates current evidence regarding the role of ultrasound, encompassing static B-mode imaging, dynamic motion analysis, contrast-enhanced techniques, and sonoelastography. Key sonographic features—such as thickening of the coracohumeral ligament, fibrosis in the axillary recess, and abnormal tendon kinematics—have been consistently associated with adhesive capsulitis and demonstrate favorable diagnostic performance. Advanced methods like contrast-enhanced ultrasound and elastography provide additional functional insights (enabling evaluation of capsular stiffness and vascular changes) which may aid in disease staging and prediction of treatment response. Despite these advantages, the clinical utility of ultrasound remains subject to operator expertise and technical variability. Limited visualization of intra-articular structures and the absence of standardized scanning protocols continue to pose challenges. Nevertheless, ongoing advances in its technology and utility standardization hold promise for the broader application of ultrasound in clinical practice. With continued research and validation, ultrasound is positioned to play an increasingly central role in the comprehensive assessment and management of adhesive capsulitis.

## 1. Introduction

Adhesive capsulitis [1,2], commonly referred to as frozen shoulder, is a clinical syndrome marked by progressive shoulder pain and significant limitation in both active and passive ranges of motion. Its prevalence in the general population ranges between 2–5% [3], while among individuals with diabetes mellitus, it increases markedly to 10–30% [4]. The condition predominantly affects adults aged 40–60, with a higher incidence reported in women compared to men [5]. Clinically, adhesive capsulitis progresses through three distinct phases: the painful “freezing” phase, the stiff “frozen” phase, and the recovery or “thawing” phase [3,6]. While spontaneous resolution occurs in some cases, long-term studies indicate that up to 50% of patients continue to experience persistent pain, stiffness, or functional impairment even several years post-onset [7]. For instance, Shaffer et al. [8] found that approximately half of the patients in their study exhibited ongoing symptoms at an average follow-up exceeding seven years (range: 2–11.75 years).

Imaging plays a central role in the diagnosis and staging of adhesive capsulitis, as well as in the differentiation from other shoulder pathologies such as rotator cuff tears, calcific tendinopathy, and glenohumeral osteoarthritis [9,10]. Although magnetic resonance imaging remains the reference standard for assessing intra-articular structures, its clinical utility may be constrained by high cost, limited availability, and longer examination times [11,12,13]. Musculoskeletal ultrasound has emerged as a valuable imaging modality due to its affordability, portability, absence of ionizing radiation, and capacity for dynamic, real-time assessment at the point of care [14,15]. Moleesaide et al. [16] demonstrated that ultrasound enables detailed visualization of key structures involved in adhesive capsulitis, including the coracohumeral ligament, rotator interval, axillary pouch, and biceps tendon sheath, while also identifying concurrent soft tissue abnormalities such as subacromial bursitis or tenosynovitis.

Recent technological advancements have further expanded the utility of ultrasound. Dynamic imaging and sonoelastography now allow for functional and mechanical assessments of soft tissues. For instance, Kato et al. [17] applied particle image velocimetry to ultrasound cine loops and identified reduced mobility of the coracohumeral ligament in patients with adhesive capsulitis. Similarly, Cheng et al. [18] used contrast-enhanced ultrasound arthrography with SonoVue to show decreased axillary recess distensibility and increased contrast leakage, providing further diagnostic distinction from normal shoulders. Moreover, several studies have described characteristic sonographic findings such as thickening of the coracohumeral ligament and hypoechoic changes in the axillary capsule [19,20]. Emerging evidence from meta-analyses supports the diagnostic accuracy of these features [21,22].

In light of these developments, a comprehensive (pictorial) review of ultrasound applications—including static and dynamic imaging techniques as well as sonoelastography—in the evaluation of adhesive capsulitis is warranted. As such, this narrative review aims to synthesize current evidence and elucidate the expanding role of ultrasound as both a diagnostic and potentially therapeutic modality in the management of this challenging shoulder disorder.

## 2. Pathogenesis

Although the precise etiology of adhesive capsulitis remains unclear, its pathogenesis is characterized by an initial phase of chronic inflammation followed by fibrosis and capsular contracture, particularly affecting the rotator interval and axillary recess [23]. Histopathological analyses have revealed dense fibroblastic proliferation, increased collagen deposition, and synovial hyperplasia within the affected capsule. In a pivotal study, tissue from the rotator interval exhibiting features akin to Dupuytren’s disease—i.e., active fibroblast proliferation, minimal inflammation, and disorganized collagen architecture—was observed [24].

At the molecular level, dysregulated cytokine signaling is thought to drive the fibrotic process. Rodeo et al. [25] identified elevated levels of transforming growth factor-beta, connective tissue growth factor, and platelet-derived growth factor in the shoulder capsule, all of which promote myofibroblast activation and extracellular matrix accumulation, leading to capsular thickening and contracture. Additionally, increased concentrations of pro-inflammatory mediators—such as interleukin-1β, tumor necrosis factor-alpha, and matrix metalloproteinases—have been detected in the synovial fluid and capsular tissue, suggesting an active inflammatory phase early in the disease course [26]. Over time, this inflammation transitions into tissue remodeling and fibrosis.

A thorough understanding of these molecular and histological changes is crucial for interpreting sonographic findings in adhesive capsulitis (including ligamentous thickening, synovial effusion, and capsular stiffness), which are discussed in the following sections.

## 3. Literature Search Strategy

Although this article is a narrative review, a structured and systematic approach was adopted to ensure inclusion of the most relevant and high-quality literature on ultrasound imaging in adhesive capsulitis. A comprehensive search of PubMed, Embase, and Scopus was conducted for articles published up to June 2025. The strategy focused on identifying original research, as well as systematic reviews and meta-analyses, that evaluated the role of both static and dynamic ultrasound imaging, including sonoelastography, in the assessment of adhesive capsulitis. Where multiple original studies addressed a given topic, results from systematic reviews or meta-analyses were prioritized and will be summarized accordingly to provide a higher level of evidence.

The search incorporated the following terms and their combinations: “adhesive capsulitis,” “frozen shoulder,” “ultrasound,” “sonography,” “dynamic ultrasound,” “sonoelastography,” and “shoulder stiffness.” Boolean operators (AND, OR) were applied to optimize sensitivity and specificity. For instance, the combination (“adhesive capsulitis” OR “frozen shoulder”) AND (“ultrasound” OR “sonography” OR “sonoelastography”) was used to identify the relevant literature. A flow diagram showing the literature selection process (Figure S1) is included in the Supplemental Materials.

Eligible studies met at least one of the following inclusion criteria: (1) documentation of sonographic structural changes such as thickening of the coracohumeral ligament or axillary pouch; (2) evaluation of the diagnostic accuracy or clinical utility of ultrasound; (3) analysis of sonoelastographic findings in the capsule or surrounding tissues; or (4) assessment of dynamic sonographic signs of movement limitation. Studies were excluded if they lacked imaging data, were unrelated to adhesive capsulitis, or consisted of non-peer-reviewed content such as conference abstracts or commentaries or were deemed less representative of the corresponding categories. The probe placement angle, set frequency, and other relevant scanning parameters for the included studies are summarized in Tables S1–S4 in the Supplementary Materials.

## 4. Static B-Mode Examination

In the realm of static B-mode ultrasound for adhesive capsulitis, recent meta-analyses reinforce its diagnostic accuracy. A 2020 systematic review and meta-analysis [21] which included seven studies comprising 446 patients (490 shoulders), evaluated the accuracy of grey-scale ultrasonography against magnetic resonance imaging or arthroscopy as reference standards. The analysis reported an overall sensitivity of 88% (95% CI: 74–95%) and specificity of 96% (95% CI: 88–99%), with a positive likelihood ratio of 23.89 (95% CI: 6.31–90.51), a negative likelihood ratio of 0.12 (95% CI: 0.05–0.29), and an area under the summary receiver operating characteristic (SROC) curve of 0.97 (95% CI: 0.96–0.98). These results underscore the high diagnostic performance of ultrasound in identifying adhesive capsulitis.

**Figure 1:**
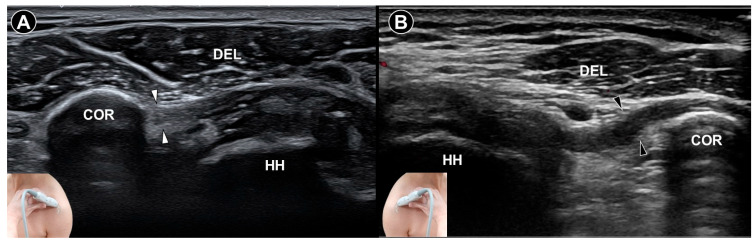
Ultrasound imaging of the coracohumeral ligament *(white arrowheads)* in a normal shoulder demonstrates a clear intra-ligamentous fibrillar pattern (**A**). In contrast, ultrasound imaging of the coracohumeral ligament *(black arrowheads)* in a shoulder with adhesive capsulitis shows thickening and loss of the intra-ligamentous fibrillar architecture (**B**). DEL, deltoid muscle; COR, coracoid process; HH, humeral head.

**Figure 2:**
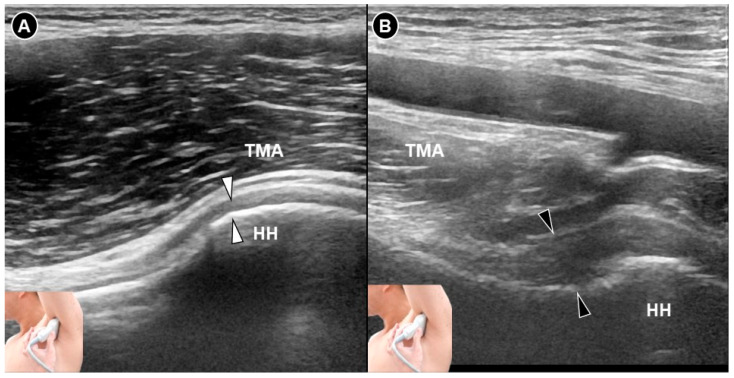
Ultrasound imaging of the inferior glenohumeral joint *(white arrowheads)* in a normal shoulder (**A**). In contrast, imaging of the inferior glenohumeral ligament *(black arrowheads)* in a shoulder with adhesive capsulitis reveals thickening of the inferior glenohumeral capsule (**B**). HH, humeral head; TMA, teres major.

**Figure 3:**
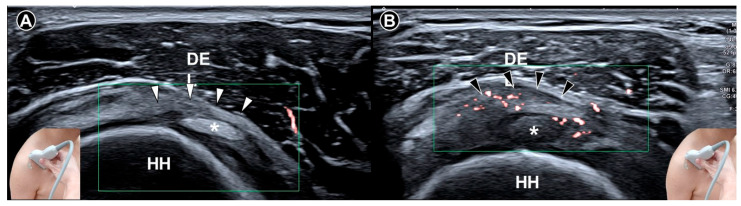
Ultrasound imaging of the rotator cuff interval *(white arrowheads)* in a normal shoulder (**A**). In contrast, the coracohumeral ligament *(black arrowheads)* in a shoulder with adhesive capsulitis exhibits increased vascularity (**B**). DEL, deltoid muscle; HH, humeral head; *asterisks*, long head tendon of the biceps brachii.

Four characteristic sonographic features were identified: coracohumeral ligament thickening (defined as a threshold of 3.0 mm) (Figure 1), inferior capsule thickening (cutoff values ranging from 2.0 mm to 3.5 mm) (Figure 2), rotator interval abnormalities (hypoechoic soft tissue thickening and/or increased vascularity) (Figure 3), and restriction of the range of motion. Their respective sensitivities were 64.4% (95% CI: 48.8–78.1), 82.1% (95% CI: 73.8–88.7), 82.6% (95% CI: 74.1–89.2), and 94.3% (95% CI: 84.3–98.8), while specificities were 88.9% (95% CI: 76.0–96.3), 95.7% (95% CI: 90.3–98.6), 93.9% (95% CI: 89.8–96.7), and 90.9% (95% CI: 75.7–98.1), respectively. The restriction of the range of motion will be further elaborated in the section on Dynamic Examination. These findings support the incorporation of B-mode ultrasound into routine diagnostic workflows for adhesive capsulitis, particularly in settings where magnetic resonance imaging is less accessible or cost-prohibitive.

Although four sonographic features—thickening of the coracohumeral ligament, thickening of the inferior capsule, abnormalities at the rotator interval, and restriction of range of motion during dynamic ultrasound—were identified in the meta-analysis by Wu et al. [21], their definitions and measurement criteria varied significantly across studies. For instance, thickening of the coracohumeral ligament was quantitatively defined in only one study [27], and the criteria used to define abnormalities at the rotator interval were inconsistently applied. These variations represent a limitation of the meta-analysis and highlight the broader lack of standardization in the ultrasound evaluation of adhesive capsulitis. In the latter part of this section, several representative studies are discussed.

Homsi et al. [19] included 17 arthrography-confirmed adhesive capsulitis cases among a cohort of 306 patients and found that mean coracohumeral ligament thickness measured by ultrasound was notably elevated at 3 mm in affected shoulders, compared to 1.34 mm in asymptomatic shoulders and 1.39 mm in other painful shoulders (all *p* < 0.05). This early evidence underpins the use of coracohumeral ligament thickening as a sonographic hallmark of the condition.

In parallel, Michelin et al. [28] conducted a retrospective ultrasound study evaluating inferior capsule thickness in 20 patients with unilateral capsular contracture confirmed either clinically or by magnetic resonance imaging. Using the axillary approach during maximal abduction, they measured the inferior glenohumeral capsule orthogonally to the inferior glenohumeral ligament. The average capsule thickness was significantly greater in affected shoulders (4.0 mm) than in asymptomatic contralateral shoulders (1.3 mm), with a *p*-value < 0.0001. These results not only reinforce the diagnostic relevance of inferior capsule thickening but also emphasize the utility of standardized measurement techniques in improving the sonographic assessment of adhesive capsulitis. This study has several limitations. It only included patients with clinically evident stiff shoulders, limiting applicability to earlier disease stages, and did not assess the reliability or measurement error of the ultrasound technique. Additionally, the monocentric design and small sample size warrant confirmation through larger prospective studies.

Regarding abnormalities in the rotator cuff, Lee et al. [29] exemplified those changes by prospectively investigating 30 individuals clinically diagnosed with adhesive capsulitis and comparing them with two control groups: 10 healthy volunteers and 100 patients with suspected rotator cuff tears. The diagnosis of adhesive capsulitis was based on clinical criteria, including shoulder pain and stiffness lasting more than 15 weeks, increasing severity at rest, and restricted motion greater than 30° in at least two planes. Using both grayscale and color Doppler ultrasound, the researchers assessed the rotator interval. Among the adhesive capsulitis group, 87% (26 patients) exhibited a hypoechoic pattern with increased vascularity in the rotator interval. Three patients showed hypoechoic changes without hypervascularity, while one had normal sonographic findings. Arthroscopic evaluation—performed in all cases—confirmed fibrovascular inflammatory soft-tissue changes in the rotator interval consistent with adhesive capsulitis. In contrast, such sonographic and arthroscopic features were absent in healthy individuals and those with suspected rotator cuff tears.

## 5. Contrast Enhanced Examination

This technique has emerged as a promising tool for evaluating adhesive capsulitis of the shoulder. Ahn et al. [30] assessed the rotator interval in five patients with unilateral adhesive capsulitis and reported a significantly higher mean peak contrast intensity in the affected shoulders (5.45 dB) compared to the unaffected side (0.72 dB; *p* < 0.05), paralleling contrast-enhanced MRI results. Cheng et al. [18] demonstrated that ultrasound arthrography using the contrast agent SonoVue significantly improved diagnostic accuracy compared to conventional ultrasound. In their comparative study of 45 patients with adhesive capsulitis and 45 matched controls, the axillary recess volume was significantly reduced in the adhesive capsulitis group (mean 1.14 mL) compared to controls (mean 1.59 mL; *p* < 0.05). Moreover, intra-articular filling defects were observed in 91% of affected shoulders, and synovitis-like abnormalities in 76%, whereas these features were absent in the control group. More recently, Cheng et al. [27] utilized contrast-enhanced ultrasound during ultrasound-guided hydrodilatation in 40 patients and achieved a first-attempt joint access success rate of 87.5% with an average injection volume of 21 mL. Contrast-enhanced ultrasound allowed for real-time visualization of capsular expansion and identified extracapsular fluid leakage in approximately 80% of patients who showed limited clinical improvement. Collectively, these findings indicate that ultrasound contrast agents such as SonoVue not only improve visualization of adhesive capsulitis-related pathology but also enhance procedural accuracy and may help predict treatment response [31].

However, it is important to note that neither of the cited studies directly compared the diagnostic performance of contrast-enhanced ultrasound with conventional grayscale or Doppler ultrasound. As such, the added diagnostic value of contrast enhancement remains uncertain. Additionally, the use of contrast agents introduces practical limitations, including higher cost, the need for intravenous or intra-articular administration, and limited applicability in routine outpatient clinical practice. These factors significantly constrain the generalizability of contrast-enhanced ultrasound and should be acknowledged as major limitations in the current literature.

## 6. Dynamic Examination

**Figure 4:**
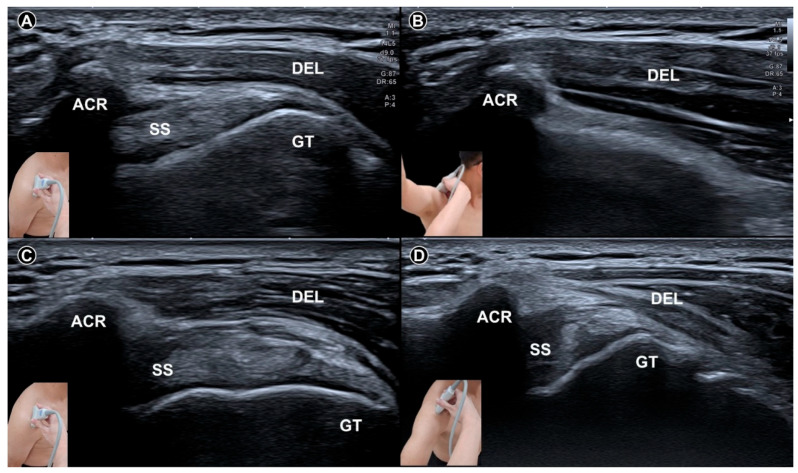
Dynamic ultrasound imaging of the subacromial region at neutral resting position (**A**) and during shoulder abduction (**B**) in a shoulder without adhesive capsulitis shows the greater tubercle of the humerus rotating beneath the acromion. In contrast, in a shoulder with adhesive capsulitis, the greater tubercle remains outside the acromion even during maximal shoulder abduction (**C**) resting; (**D**) maximal abduction. The figure was redrawn by the authors with reference to Ryu et al. [33]. ACR, acromion; SS, supraspinatus tendon; GT, greater tubercle; DEL, deltoid muscle.

Dynamic ultrasound has been widely explored for its diagnostic potential in evaluating adhesive capsulitis, particularly due to its ability to capture movement limitations in real time [32]. Ryu and colleagues [33] focused on identifying restricted sliding of the supraspinatus tendon beneath the acromion during shoulder elevation (Figure 4). During the examination, the sonographer stabilized the patient’s elbow and used a longitudinal transducer placement over the supraspinatus tendon to visualize its motion as the arm was abducted. Two key sonographic findings were proposed as indicators of adhesive capsulitis: (1) persistent restriction of the tendon’s gliding motion and (2) uninterrupted visualization of the supraspinatus tendon throughout arm elevation (Video S1, normal shoulder; Video S2, adhesive capsulitis). The diagnostic performance of these ultrasound findings was validated against arthrography, which served as the reference standard. Among the 23 patients with arthrographically confirmed adhesive capsulitis, 21 demonstrated the sonographic criteria of adhesive capsulitis, resulting in a sensitivity of 91%, specificity of 100%, and overall diagnostic accuracy of 92%. These findings underscore the value of dynamic ultrasound in detecting functional limitations associated with adhesive capsulitis, particularly involving the supraspinatus tendon. However, it should be noted that the study did not include a dedicated control group. All enrolled subjects were clinically suspected of having frozen shoulders, and the specificity values were based only on those not meeting the arthrographic criteria. This limits the generalizability of the findings, as the proposed ultrasound features may not be entirely specific to adhesive capsulitis and could potentially be observed in other shoulder pathologies. This limitation should be taken into account when interpreting the diagnostic utility of dynamic ultrasound in clinical practice.

**Figure 5:**
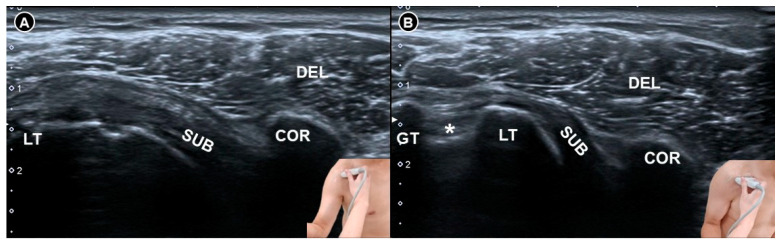
Dynamic ultrasound imaging of the subcoracoid region during shoulder external rotation. In a normal shoulder (**A**), the subscapularis tendon is fully visualized whereas in a shoulder with adhesive capsulitis (**B**), only the distal portion of the tendon is visible and appears hypoechoic due to anisotropy, as the tendon fibers are not perpendicular to the ultrasound beam. The figure was redrawn by the authors with reference to Tandon et al. [34]. DEL, deltoid muscle; COR, coracoid process; SUB, subscapularis tendon; LT, lesser tubercle; GT, greater tubercle; asterisk, long head tendon of the biceps brachii.

Tandon and colleagues [34] evaluated 90 individuals—comprising 30 patients with adhesive capsulitis, 30 with nonspecific shoulder pain, and 30 healthy controls—to compare dynamic sonographic findings with clinical evaluation and magnetic resonance imaging. Two experienced sonographers independently assessed the restriction of abduction and external rotation. Their results showed that restriction of external rotation (Figure 5) was a highly specific indicator of adhesive capsulitis, with a sensitivity of 86.2% and a specificity of 92.8%. In contrast, restriction of abduction was found to be nonspecific, with a specificity of only 6.7%. These findings suggest that dynamic assessment of external rotation is more reliable than that of abduction for diagnosing adhesive capsulitis.

**Figure 6:**
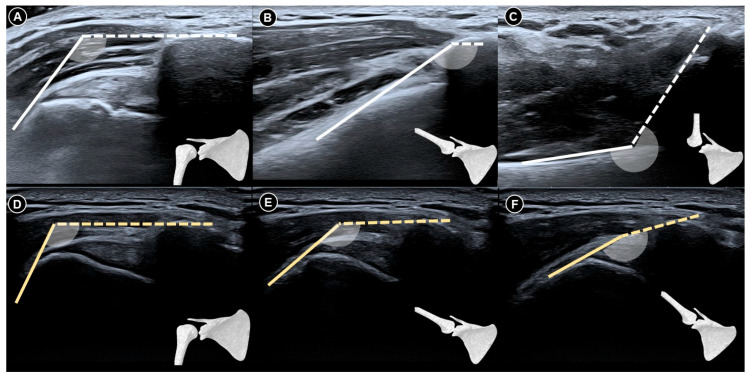
Dynamic ultrasound images of a normal shoulder captured at neutral resting position (**A**), during abduction beyond 90 degrees (**B**), and at full elevation to 180 degrees (**C**). Corresponding images of a shoulder with adhesive capsulitis at rest (**D**), abduction past 90 degrees (**E**), and maximal abduction (**F**). The figure was redrawn by the authors with reference to Chang et al. [35].

Recently, Chang et al. [35] introduced the humerus-acromion angle model to assess adhesive capsulitis severity by quantifying scapulohumeral rhythm during shoulder abduction. Normally, it follows a 2:1 ratio—every 3° of elevation involves 2° at the glenohumeral joint and 1° from scapular rotation. During ultrasound, positioning the probe along the acromion, while the patient abducts the arm, reveals a progressive angle increase between the acromial cortex and the humerus, ranging from ~120° at rest to ~240° at full abduction. This change reflects both glenohumeral rotation and inferior humeral translation. In frozen shoulder, this angle plateaus prematurely despite passive elevation, indicating impaired glenohumeral motion. Further abduction then depends on limited scapular rotation (~60°). This model offers a more precise means to evaluate mechanical constraints in glenohumeral adhesive capsulitis (Figure 6).

## 7. Sonoelastography

This is an advanced ultrasound technique that quantifies tissue stiffness and has emerged as a promising modality for evaluating adhesive capsulitis. By providing both qualitative and quantitative insights into the biomechanical properties of the shoulder capsule and surrounding soft tissues, it complements conventional B-mode ultrasound. Further, sonoelastography offers functional information that may aid in staging the disease and guiding treatment planning.

**Figure 7:**
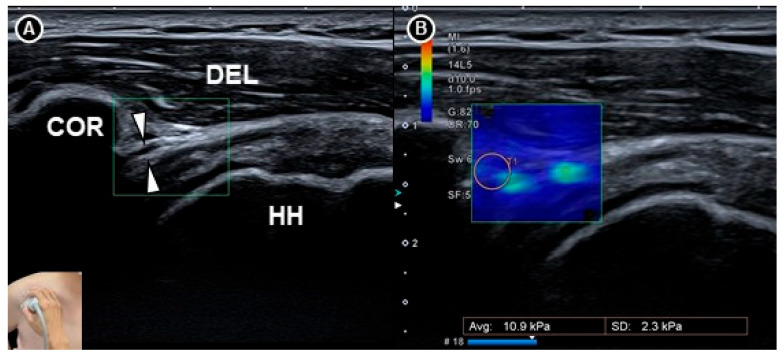
Shear wave sonoelastography of the coracohumeral ligament *(white arrowheads)* shown in grayscale imaging (**A**) and color elastogram (**B**). The figure was redrawn by the authors with reference to Wu et al. [36]. DEL, deltoid muscle; COR, coracoid process; HH, humeral head.

Wu and colleagues [36] examined the elasticity of the coracohumeral ligament using shear wave elastography in 20 patients with clinically suspected adhesive capsulitis and 30 healthy individuals (Figure 7). Measurements of ligament thickness and stiffness were taken from both shoulders in the neutral position and under maximal external rotation. Among the patients, the symptomatic shoulders showed significantly greater coracohumeral ligament thickness (*p* < 0.001) and a higher elastic modulus in the neutral position (median 234.8 kilopascals, interquartile range 174.4 to 256.7) compared to the asymptomatic side (median 203.3 kilopascals, interquartile range 144.1 to 242.7; *p* = 0.004). However, this difference was not statistically significant under maximal external rotation (*p* = 0.123).

**Figure 8:**
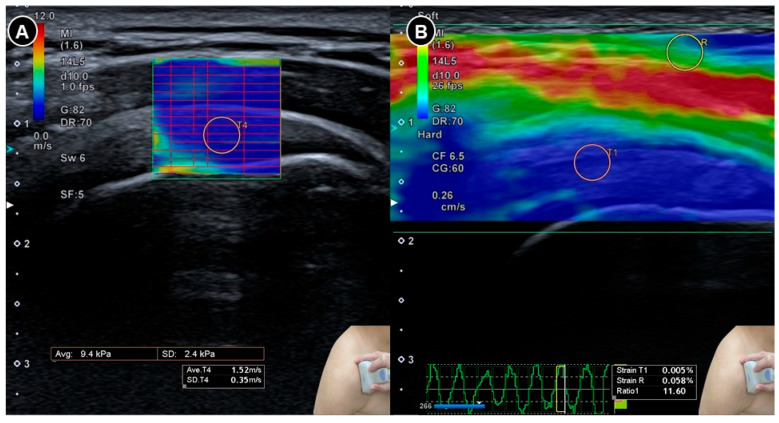
Sonoelastography of the supraspinatus tendon. Shear wave elastography (**A**) demonstrates the shear wave speed and strain elastography (**B**) illustrates the strain ratio. The figure was redrawn by the authors with reference to Yun et al. [37].

Yun and colleagues [37] conducted a prospective case-control study involving 25 symptomatic shoulders from 20 patients with adhesive capsulitis and 24 asymptomatic shoulders from 18 individuals as controls. Both shear wave and strain elastography were performed on the supraspinatus and infraspinatus tendons. In patients with adhesive capsulitis, the mean velocity and stiffness were significantly higher (*p* < 0.001), while the strain ratio (defined as strain in the subcutaneous fat divided by strain in the target tendon) was significantly lower (*p* < 0.001) than in the control group (Figure 8). Receiver operating characteristic curve analysis showed excellent diagnostic accuracy, with area under the curve values exceeding 0.970 for both tendons. Subsequently, Chang et al. [38] raised concerns regarding the interpretation of the strain ratio whereby they pointed out that in normal physiology, tendons are stiffer than subcutaneous fat and therefore exhibit less strain, which should result in a strain ratio greater than 1. However, the reported median strain ratio of the supraspinatus tendon in the control group was 0.38, suggesting an inconsistency in either the calculation or interpretation of the strain values. This discrepancy underscores the need for standardized reporting conventions in elastography studies.

Chiu and colleagues [39] published a systematic review and meta-analysis that included 11 cross-sectional studies. They found a trend toward increased stiffness in the supraspinatus and infraspinatus tendons in patients with adhesive capsulitis, with standardized mean differences of 2.103 (95% confidence interval, –0.151 to 4.357; *p* = 0.067) and 1.548 (95% confidence interval, –0.032 to 3.127; *p* = 0.055), respectively.

Although sonoelastography is still a relatively new tool in musculoskeletal imaging, the aforementioned early findings suggest that it may allow for earlier detection of pathological stiffness, improve staging accuracy, and support more individualized treatment approaches in patients with adhesive capsulitis. However, its routine use in clinical practice will require further validation through studies with standardized acquisition protocols and normative reference data.

## 8. Clinical Utility and Limitations

In clinical settings, ultrasound serves as a valuable tool for evaluating structural changes associated with adhesive capsulitis. Key sonographic features include thickening of the coracohumeral ligament, fibrosis within the axillary pouch, and abnormal tendon motion patterns. The dynamic capabilities further enable real-time assessment during shoulder movement, which can aid in differentiating adhesive capsulitis from other conditions presenting with pain and stiffness, such as subacromial impingement or rotator cuff disorders.

In addition to its diagnostic utility, ultrasound increasingly supports therapeutic interventions for adhesive capsulitis. Image-guided techniques improve the precision of intra-articular corticosteroid injections and hydrodilatation procedures [40,41,42,43] intended to stretch the contracted joint capsule. Real-time targeting of specific regions, such as the rotator interval or axillary recess, may enhance the accuracy and comfort of these interventions. Emerging applications, including elastographic measurement of capsular stiffness, hold potential for tailoring treatment (based on disease severity or progression), though such uses remain largely investigational.

In our review of the sonoelastography section, Wu et al. [36] reported that patients with adhesive capsulitis exhibited increased coracohumeral ligament thickness and stiffness, as measured by shear wave elastography, particularly in the neutral shoulder position. Similarly, Yun et al. [37] employed both shear wave and strain elastography on the rotator cuff tendons and found significantly higher stiffness and lower strain ratios in affected shoulders, reflecting altered biomechanical properties. However, as highlighted by Chang et al. [38], inconsistencies in the interpretation of strain ratios—such as the unexpectedly low values reported for control subjects—emphasize the need for standardized methodologies. Collectively, these findings support the clinical utility of sonoelastography while reinforcing the importance of consistent reporting protocols to enable reliable comparisons across studies.

Furthermore, ultrasound can help differentiate true adhesive capsulitis from other conditions with overlapping symptoms, such as subacromial impingement syndrome [44,45]. In cases of adhesive capsulitis, the glenohumeral capsule may demonstrate regionally uneven thickening. Ultrasound-guided hydrodilatation [46,47,48,49] can be tailored to specifically target these affected areas [50]. For example, our review highlights that thickening of the coracohumeral ligament—which is connected to the anterior glenohumeral capsule—is a common finding in adhesive capsulitis and may warrant more focused intervention.

Despite its advantages, a major limitation in the current literature on ultrasound for adhesive capsulitis is the lack of standardized definitions for sonographic features. Studies frequently adopt varying diagnostic criteria and focus on different anatomical landmarks, leading to inconsistencies in interpretation and limited comparability. For example, while some reports highlight coracohumeral ligament thickening or axillary pouch fibrosis, the threshold values and measurement techniques vary considerably across investigations. This heterogeneity hampers the development of universally accepted protocols and undermines the reproducibility of ultrasound-based diagnoses.

Beyond definitional inconsistencies, several methodological limitations remain insufficiently addressed. These include the influence of individual patient anatomy, the high cost and potential adverse effects of contrast agents used in certain studies, and concerns regarding the overall cost-effectiveness of these imaging approaches. The operator-dependent nature of ultrasound—where diagnostic accuracy is strongly influenced by the physician’s skill and experience—further contributes to variability in findings. Additionally, many studies involve small sample sizes without adequate justification, limiting statistical power and generalizability. In some cases, the absence of a control group further weakens the strength of the conclusions, and the rationale for this omission should be explicitly stated to aid interpretation. Establishing consensus definitions and standardized imaging protocols—ideally through multicenter studies, expert panel recommendations, and rigorous methodological design—will be crucial for improving diagnostic reliability and promoting broader clinical adoption.

## 9. Conclusions

In addition to its ability to detect key structural abnormalities, recent advances in B-mode static imaging, dynamic assessment, contrast-enhanced ultrasound, and sonoelastography have upscaled our insight into the biomechanical and pathological features of adhesive capsulitis. Although limitations such as operator dependency and limited visualization of intra-articular components persist, ultrasound remains a highly accessible and increasingly sophisticated modality. Ongoing efforts to standardize imaging protocols and validate novel techniques will be critical to fully realize its diagnostic and therapeutic potential in the management of this condition.

## Data Availability

No new data were created or analyzed in this study. Data sharing is not applicable to this article.

## Supplementary Materials

The following supporting information can be downloaded at: https://www.mdpi.com/article/10.3390/diagnostics15151924/s1, Figure S1: Flow Diagram of Literature Search; Table S1: Integrated Summary of Ultrasound Scanning Parameters in Adhesive Capsulitis: Static B-Mode Examination; Table S2: Integrated Summary of Ultrasound Scanning Parameters in Adhesive Capsulitis: Contrast Enhanced Examination; Table S3: Integrated Summary of Ultrasound Scanning Parameters in Adhesive Capsulitis: Dynamic Examination; Table S4: Integrated Summary of Ultrasound Scanning Parameters in Adhesive Capsulitis: Sonoelastography; Video S1: Dynamic ultrasound examination in a normal shoulder; Video S2: Dynamic ultrasound examination in a shoulder with adhesive capsulitis.
