# Supplementary material for: Ultrasound in Adhesive Capsulitis: A Narrative Exploration from Static Imaging to Contrast-Enhanced, Dynamic and Sonoelastographic Insights
Source: Diagnostics (Basel). 2025 Jul 31;15(15):1924. doi: 10.3390/diagnostics15151924 (PMC12346586; doi:10.3390/diagnostics15151924)
Supplement: Supplementary file 1 [file diagnostics-15-01924-s001.zip › Supplemental Figure and Table.pdf]

Figure S1. Flow Diagram of Literature Search

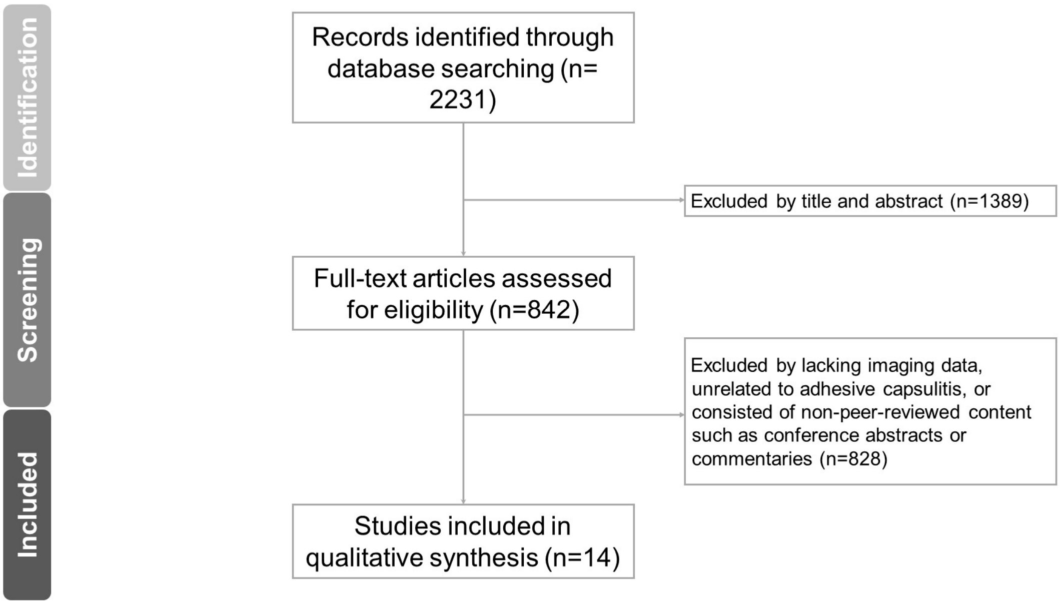

Table S1. Integrated Summary of Ultrasound Scanning Parameters in Adhesive Capsulitis: Static B-Mode Examination

Study	Probe Placement	Shoulder Position	Transducer Frequency	Use of Doppler Imaging	Additional Notes
Homsi et al. (2006)	Oblique axial view over the coracoid process and sagittal view after 90-degree rotation	Neutral position with the forearm extended; dynamic shoulder rotation (external and internal) used to identify the coracohumeral ligament	5 to 13 megahertz using ATL HDI 5000, Siemens Sonoline Elegra, and GE Logic 700	Not used	Measurements of coracohumeral ligament performed in both axial and sagittal planes; anisotropy highlighted; care taken to differentiate from pectoralis minor anatomical variants
Michelin et al. (2013)	Axillary region, orthogonal	Maximal shoulder abduction	5 to 18 megahertz using	Not used	Coronal plane used to ensure tension in the inferior glenohumeral

	orientation to the inferior glenohumeral ligament, primarily in the axial plane	with neutral rotation	Siemens S2000 and Toshiba Aplio XG systems		ligament; anisotropy was utilized to optimize probe angle for maximum echogenicity; bilateral measurements performed symmetrically
Lee et al. (2005)	Oblique plane over the anterior shoulder targeting the rotator interval	Patient's fist held beside the trunk	8 to 15 megahertz using a Siemens Acuson Sequoia 15L8 transducer	Color Doppler was used	Assessed hypoechoic soft tissue and vascularity at the rotator interval; diagnosis confirmed by consensus between a musculoskeletal radiologist and a trained sonographer

Table S2. Integrated Summary of Ultrasound Scanning Parameters in Adhesive Capsulitis: Contrast Enhanced Examination

Study	Probe Placement	Shoulder Position	Transducer Frequency	Use of Doppler Imaging	Additional Notes
Ahn et al. (2016)	Oblique sagittal plane over the rotator interval	Patient seated with elbow flexed and shoulder extended	5 to 12 megahertz using Philips iU22 system	Contrast-specific low mechanical index mode (0.08), not standard Doppler	2.4 mL SonoVue injected intravenously followed by 5 mL saline flush; time-intensity curves analyzed (delay time, time-to-peak, peak intensity, rate-of-rise); enhancement matched magnetic resonance imaging findings
Cheng et al. (2024)	Posterior approach with the transducer placed along the long axis of the infraspinatus tendon, needle	Patient lying in lateral decubitus position with the affected shoulder side up	3 to 9 megahertz using Philips EPIQ 7 system	Contrast-enhanced ultrasound used for real-time monitoring, not standard Doppler	22-Gauge needle used; maximum injection volume was 23 mL; SonoVue contrast agent used; procedure terminated when plunger resistance was felt or patient experienced pain; leakage graded (none, minimal, massive);

	inserted in-plane from lateral to medial				capsule rupture confirmed with magnetic resonance imaging

Table S3. Integrated Summary of Ultrasound Scanning Parameters in Adhesive Capsulitis: Dynamic Examination

Study	Probe Placement	Shoulder Position	Transducer Frequency	Use of Doppler Imaging	Additional Notes
Ryu et al. (1993)	Longitudinal over supraspinatus tendon for dynamic assessment	Neutral shoulder followed by lateral arm elevation during real-time observation	5 megahertz linear phased-array transducer (Ultramark 9)	Not used	Continuous visualization of supraspinatus tendon during lateral elevation assessed for motion restriction; criterion was incomplete sliding under the acromion
Tandon et al. (2017)	Oblique axial for coracohumeral ligament; oblique coronal for supraspinatus; medial longitudinal for subscapularis	Patient seated with neutral shoulder and extended forearm for coracohumeral ligament; dynamic abduction and external rotation for dynamic assessment	5 to 12 megahertz using Philips HDI 5000 or Sonoline Antares	Yes, color Doppler was used for vascularity in rotator interval	Coracohumeral ligament measured with arm in external rotation; both static (ligament thickness, rotator interval soft tissue) and dynamic (abduction and external rotation restriction) parameters evaluated

Table S4. Integrated Summary of Ultrasound Scanning Parameters in Adhesive Capsulitis: Sonoelastography

Study	Probe Placement	Shoulder Position	Transducer Frequency	Use of Doppler Imaging	Additional Notes
Wu et al. (2016)	Axial oblique plane over the lateral border of the coracoid process to	Supine position, elbow flexed at 90°, shoulder in neutral and	4 to 15 megahertz using Supersonic Imagine	Not used; shear-wave elastography	Transducer gently placed with gel layer (no compression); breath-holding requested to reduce motion artifacts;

	visualize the coracohumeral ligament	maximal external rotation	system	applied	region of interest set 2 mm from coracoid process; elasticity measured in kilopascals to assess coracohumeral ligament stiffness in neutral and externally rotated positions
Yun et al. (2019)	Oblique coronal plane at the distal supraspinatus and infraspinatus tendons near the greater tuberosity	Seated position, shoulder in neutral (no abduction, adduction, or rotation), elbow extended	LOGIQ E9 system (GE Healthcare); shear-wave and strain elastography modes	Not used; both shear-wave and strain ultrasound elastography applied	Assessed elasticity using mean/maximum/minimum velocity and stiffness for shear-wave elastography and strain ratio for strain elastography; regions of interest placed 0.5–1 cm from the greater tuberosity; stiffer tendons found in patients with adhesive capsulitis compared to controls
